# Poleward range expansion without a southern contraction in the ground beetle *Agonum viridicupreum* (Coleoptera, Carabidae)

**DOI:** 10.3897/zookeys.100.1535

**Published:** 2011-05-20

**Authors:** Claudia Drees, Pietro Brandmayr, Jörn Buse, Petra Dieker, Stephan Gürlich, Jan Habel, Ingmar Harry, Werner Härdtle, Andrea Matern, Hartmut Meyer, Roberto Pizzolotto, Markus Quante, Katharina Schäfer, Andreas Schuldt, Angela Taboada, Thorsten Assmann

**Affiliations:** 1 Tel Aviv University, George S. Wise Faculty of Life Sciences, Department of Zoology, The National Collections of Natural History, Tel Aviv 69978, Israel; 2 Università della Calabria, Dipartimento di Ecologia, I-87036 Arcavacata di Rende (CS), Italy; 3 Johannes Gutenberg-University Mainz, Institute of Zoology, Department of Ecology, Becherweg 13, D-55099 Mainz, Germany; 4 Musée National d’Histoire Naturelle, Section Zoologie des Invertébrés, L-2160 Luxembourg, Luxembourg; 5 Verein für Naturwissenschaftliche Heimatforschung zu Hamburg e.V., Martin-Luther-King-Platz 3, D-20146 Hamburg, Germany; 6 Leuphana University Lüneburg, Institute of Ecology and Environmental Chemistry, Scharnhorststr. 1, D-21335 Lüneburg, Germany; 7 ABL, Nägeleseestraße 8, D-79102 Freiburg, Germany; 8 GKSS Research Center, Institute for Coastal Research, Max-Planck-Straße 1, D-21502 Geesthacht, Germany; 9 Area of Ecology, Department of Biodiversity and Environmental Management, University of León, Campus de Vegazana s/n, E-24071 León, Spain

**Keywords:** chronosequence, climate change, distribution area, global change, wetlands, power of dispersal, migration, range shift

## Abstract

We investigated the extent of poleward shifts in the distribution range of *Agonum viridicupreum* due to climate change in the western Palaearctic. Species’ records were obtained from extensive literature sources as well as from collections, and consistent amateur entomologists’ recordings. Within the general geographic range of the species, we analyzed in detail two parts of both, the northern and southern distribution range boundaries: (1 and 2) north-western Germany (leading or high-latitude edge), (3) Israel and (4) southern Italy (rear or low-latitude edge). Temporal changes in the occurrence data of the species indicated a northward shift of the leading edge of a minimum of 100 km within the last 50 to 100 years. In contrast, according to the data gathered, the rear edge has not changed during the last decades. Further studies are needed in order to fully understand the underlying mechanisms of the different behaviour of leading and rear range edges of *Agonum viridicupreum* in the current context of global change. Despite our incomplete understanding, chronosequences of the occurrence of the given species have the potential to optimize climate niche modelling to predict trends in the distribution range in the future.

## Introduction

For about 250 years, man has released radiatively active gases and particles in substantial amounts into the atmosphere. As one of the consequences, the global mean near surface temperature has increased, a phenomenon commonly referred to as ‘global warming’ or ‘climate change’. Deduced mainly from instrumental observations initiated around 1860, the observed climate change can be attributed to a large extent to human activities, which influence not only global temperature, but also pH-values of the oceans, precipitation and the general hydrological cycles on Earth (IPCC 2007; Quante 2010).

For many animal and plant species, theoretical analyses on the climate determination of the species’ occurrence have predicted a general poleward shift and (in mountain areas) an uphill shift of the given distribution areas and populations, respectively, as a response to climate change. In agreement with theory, numerous range shifts have been documented in the last years. Examples are known from vascular plants, birds, and many insects such as butterflies, dragonflies and damselflies (Hickling et al. 2006; Parmesan 2006; Pauli et al. 2007). Carabid beetles and other epigean soil invertebrates are well known as highly dynamic colonizers of glacier forelands in the last two centuries, and uphill shifts of several hundred metres altitude have been described in the Austrian and Italian Alps and for Scandinavian mountains (Gobbi et al. 2006; Gobbi et al. 2007).

Poleward shifts of distribution areas are very likely also for widely distributed carabid species (in contrast to species with restricted distribution areas, i.e. endemics), as their patterns of geographic distribution are strongly determined by climatic factors (as shown by a large-scale analysis of West Palaearctic ground beetle diversity, Schuldt and Assmann 2009). Indeed, northward shifts of ground beetle species have been documented several times in the literature. Already Lindroth (1972), certainly the most important carabid biogeographer, demonstrated that several species, especially those with flight activity, have expanded their distribution areas northwards in Fennoscandia since the middle of the last century. Some of these species went on spreading polewards, e.g. *Stenolophus mixtus* in Scandinavia (Kvamme 1978; Palm 1982) or in Great Britain (Blake 2001).

Moreover, ground beetles with their northern distribution limit in Britain have moved about 50 km northwards within a period of about two decades (Hickling et al. 2006). Further examples of poleward shifts in the geographic distribution of carabids can be obtained from the faunistic literature throughout Europe, e.g. *Demetrias imperialis* in countries around the Baltic Sea (Silfverberg 2005), and *Tachyta nana*, *Diachromus germanus*, and *Acupalpus luteatus* in north-western German lowlands (Ziegler 2004). Besides, expansions of carabid species’ distribution areas are conspicuous and numerous amateur entomologists consistently notify new records.

However, previous studies on poleward range margin shifts of ground beetles have mainly focused on the leading (i.e. current high altitudinal and latitudinal) edges of their distribution areas (literature cited above). Changes occurring at the leading edge are interesting, especially in the framework of dispersal biology, and they enable us to understand many population biological processes (Hengeveld 1985; 1989). In contrast, despite the fact that leading edges seem to be more relevant than rear (i.e. current low altitudinal and latitudinal) edges, the latter may be of greater importance for the long-term survival of species (Hampe and Petit 2005). This is related to the different histories of leading and rear edges. In general, at the poleward limits of distributions newly founded populations are recent and, therefore, only short-term adaptations have been possible. In contrast, many of the rear edge populations are close to their glacial refuges, i.e. the specimens are genetically more variable and, thus, allow greater power of adaptability and preadaptation (Hampe and Petit 2005).

Nevertheless, up to now, there is no available study comparing the reaction of a ground beetle species at both margins of its distribution range. Thus, in this study we aimed at investigating the extent of poleward range shifts at both the leading and rear edges of the distribution area of a carabid species due to recent climate changes. We selected *Agonum viridicupreum* as our study object because it fulfils many preconditions of a suitable model species to assess potential margin shifts: The specimens can be easily found in the field, they are fully winged and fly actively, and the species’ habitat preferences are well-known. Furthermore, the specimens are nicely coloured, stimulating many amateur entomologists to record the species, and, therefore, allowing suitable faunistic data from large parts of its distribution area. Moreover, the species is not restricted to habitats that are influenced or even destroyed by other drivers of global change, nor have been altered simultaneously by the temperature increase in the last decades (e.g. oligotrophic peat bogs affected by increased atmospheric nitrogen depositions due to pollution, Bobbink et al. 1998).

## Material and methods

### The study species

*Agonum viridicupreum* (Goeze, 1777) is a macropterous and thermophilous species restricted to open, wet habitats such as meadows, fens and rain ponds. The day-active beetle prefers sun-exposed muddy sites where it can be easily detected by its green-bronze-coloured surface. Due to its occurrence in floodplain areas (with high probability of diversion), the dispersal of individuals is not only determined by the species’ ability to fly. Specimens can be transported downstream by flooding events into areas where the species might not be able to establish autochthonous populations (Bonn 2000; Turin 2000; personal observations).

In the Levant (Middle East, see below), the beetle lives in wet habitats, mostly close to winter or rain ponds (personal observations). In southern Italy (Calabria) the species lives in river bank habitats around *Typha* swamps or in other wet vegetation types and crops, and on lake shores, until about 1400 m above sea level.

### Distribution area and temporal changes

We reviewed the available faunistic literature for the western Palaearctic (Europe, the Mediterranean area) to determine the general distribution area of the study species (Horion 1941; Jeannel 1941f; Antoine 1955f; Kocher 1963; Magistretti 1965; Bonadona 1971; Burakowski et al. 1973f; Alfieri 1976; Bangsholt 1983; Lindroth 1985; Jeanne and Zaballos 1986; Hieke and Wrase 1988; Marggi 1992; Zaballos and Jeanne 1994; Guérguiev and Guérguiev 1995; Kryzhanovskij et al. 1995; Hurka 1996; Machard 1997; Köhler and Klausnitzer 1998; Casale and Vigna-Taglianti 1999; Drovenik and Peks 1999; Neculiseanu and Matalin 2000; Turin 2000; Marggi and Luka 2001; Bousquet 2003; Serrano 2003; Müller-Motzfeld 2004; Brandmayr et al. 2005; Curcic et al. 2007; Luff 2007; Austin et al. 2008; Desender et al. 2008) and of *Agonum fulgidicolle* Erichson, 1841, an allopatric sibling taxon of *Agonum viridicupreum* (ranked by some authors as a subspecies, e.g. Puel 1938), which occurs in north-western Africa.

The situation of faunistic recordings is sufficient for one region at the northern distribution edge (north-western Germany) and for two regions at the southern distribution edge (Levant in the Middle East, mainly Israel, and Calabria in southern Italy).

– North-western Germany has been studied by numerous amateur entomologists who have greatly contributed to our knowledge on the geographic distribution of carabid beetles. We therefore analyzed the changes in the species’ distribution separately for (a) West Lower Saxony (west of river Weser) and for (b) East Lower Saxony (east of river Weser), Hamburg, and Schleswig Holstein. For these regions records from three periods (before 1950, between 1951 and 1980, after 1980) were summed up to document tendencies in the numbers of catches.

– For Israel, the first records date from the 1920s (the beginning of modern zoological exploration of the given region by local scientists, in former times only explorers from abroad collected beetles there). We therefore distinguished only two periods of collecting: before 1980 and after 1980.

– For southern Italy (Calabria) there are scarce historical records (before 1980). However, after 1980, intensive ecological surveys were carried out on populations in several sites of the Crati river valley (Mazzei et al. 2010).

Consequently, within the global distribution area of the study species, we analyzed the northern and southern range boundaries by studying in detail the three above mentioned concrete margin regions, where the coverage of the faunistic recordings is amply and sufficient: (1 and 2) a part of the leading edge (north-western Germany: Lower Saxony, Hamburg and Schleswig-Holstein, divided into regions west and east of the river Weser), and (3 and 4) the only continental areas at the rear edge that are not limited by the sea or by the presence of *Agonum fulgidicolle* (the Levant in the Middle East: mainly Israel, and Calabria, (southern Italy)). For these areas we compiled numerous faunistic records mostly published in local journals (Westhoff 1881, 1882; Bodenheimer 1937; Barner 1954; Lohse 1954; Gersdorf and Kuntze 1957; Assmann and Ehrnsberger 1990; Assmann and Ehrnsberger 1990; Angelini 1991; Assmann 1991; Mossakowski 1991; Gürlich et al. 1995; Handke 1995; Handke and Kundel 1996; Bonn et al. 1997; Fuellhaas 1997; Nitzu 1997; Ziegler 1997; Fischer et al. 1998; Bonn 2000; Hannig and Schwerk 2000; Hannig 2001; Bonn et al. 2002; Assmann et al. 2003; Hannig 2004; Günther and Assmann 2005; Hannig 2005, 2008; Wrase 2009; Mazzei et al. 2010). At the leading edge, Assmann and Ehrnsberger (1990) as well as Irmler and Gürlich (2004) have previously observed an enlargement of the distribution range in northern Germany. Moreover, we also incorporated in our data base the species’ records obtained from museums and private collections (collections of several authors and David Wrase, Berlin (CWB), the Collection Assmann Bleckede (CAB), The National Collection of Natural History of the Tel Aviv University (TAU)) and data bases available on the internet (mainly www.entomologie.de/hamburg/karten/%0bfhl_02/_agovir1.htm and www.eurocarabidae.de). Generally, several specimens from identical dates and locality are regarded only as one record.

### Climate changes in the regions of interest

We surveyed climatological literature and compiled information about recent climate changes in the three regions north-western Germany, Israel and southern Italy (Calabria). We focussed only on changes in temperature and precipitation, the main factors influencing the ground beetles’ biology and distribution.

## Results

### Climate changes

North-West Germany – warmer springs with wetter winters and drier summers

Over the last 150 years a considerable increase of the global mean **temperature** by about 0.8°C has been observed. Also for western Europe the measurements show a warming trend. For Germany during the 20th century a mean temperature rise of about 1.0°C was reported by Schönwiese and Janoschitz (2008). This warming is not homogenous; there are noticeable seasonal and regional differences. In the western part of northern Germany a linear trend value for the temperature between 0.6°C and 0.8°C appears to be typical. For the period from 1951 to 2000 this linear trend value is slightly higher and comes close a 1°C with a tendency of marginally higher values towards the south-east. The increase in winter temperatures was higher than that for the summer. For the last decades the strongest warming was found to appear in spring. An evaluation of station data for different states in northern Germany using a different averaging method came to the conclusion that during the 20th century the mean temperature in Lower Saxony rose by 1°C, in Schleswig Holstein by 0.8°C and in the metropolitan region of Hamburg by 1.1°C (I. Meinke, GKSS pers. comm.). For the Hamburg area Schlünzen et al. (2010) report an increase in the decadal warming rate, which underlines that the temperature trend was significantly larger in the last three decades. The corresponding rates from a piecewise linear trend evaluation are 0.07 K/decade for 1891–2007, 0.19 K/decade for 1948–2007 and 0.60 K/decade for 1978–2007. Recently the strongest warming appeared in the winter months. A comparison of mean temperatures for the first and last decade of the 20th century suggests that the region in Lower Saxony west of the river Weser faced a slightly higher warming than the eastern part. This result is in conflict with the pattern shown by Schönwiese and Janoschitz (2008) and probably due to the method of comparing only two decades.

Linear 20th century **precipitation** trends for Germany have been reported to be about 8.5% (an increase from 750 mm to 800 mm, Schönwiese and Janoschitz 2008). However, because of a strong interannual variability this trend is not statistically significant. Over this period especially the winter precipitation increased, while for the summer months a decrease was observed. This increase in winter precipitation and decrease in summer precipitation was also reported for the western part of northern Germany. An evaluation of station data for different states in northern Germany using a different averaging method came to the conclusion that during the 20th century precipitation in Lower Saxony increased by about 10%, in Schleswig Holstein by about 12.5% and in the metropolitan region of Hamburg by about 12% (I. Meinke, GKSS pers. comm.). For the Hamburg area Schlünzen et al. (2010) report a significant increase in precipitation rate. The corresponding rates from a piecewise linear trend evaluation are ~0.8 mm/year for 1891–2007 and 1.3 mm/year for 1948–2007. The increase again is most pronounced for the winter months. For the months April and July in the period between 1978 and 2007 a significant decrease in precipitation in the Hamburg area has been found.

### Levant (Israel): warmer and drier in the north, wetter in the south

An analysis for the period 1964 to 1994 of **temperature** measurements at 40 stations evenly distributed over Israel came to the conclusion that there appears to be a general warming trend, with some local exceptions, i.e. in the south, which could be related to enhanced aerosol emission (Ben-Gai et al. 1999). This general trend has been confirmed by a more recent reanalysis study (Saaroni et al. 2003); this study also notes that for the last decades July replaces August as the warmest month of the year. The overall analysis reveals a complex change pattern. First, the summers have become warmer, while the winters became colder; second, there exists a significant decreasing trend of the daily maximum and minimum temperature during the cool season and an increasing trend during the warm season (Ben-Gai et al. 1999).

Concerning climatological **precipitation** trends the Levant has to be divided into a southern and northern part. An analysis of winter half-year precipitation over the entire Mediterranean region reveals predominating rainfall decreases during the last 50 years. The areas deviating from this general trend includes southern Israel (Jacobeit et al. 2007). Several studies report opposing trends of annual rainfall for the eastern Mediterranean (e.g. Steinberger and Gazit-Yaari 1996; Jacobeit et al. 2007; Khatib et al. 2007), a decrease of rainfall amounts in the northern part of Israel and increase for southern regions during recent decades. There are indications that the observed trend differences are the outcome of changes in synoptic conditions in the eastern Mediterranean region (Steinberger and Gazit-Yaari 1996). In the overall series of wettest winters (see above, analysis by Luterbacher et al. 2006) the southern part of the Levant was slightly drier than the climatological mean and in the overall driest winter series this region was wetter than the 1961 to 1990 average (Luterbacher et al. 2006). For the northern part of the Levant the trends seem to be *vice versa*; consistently different trend behaviour in the southern part compared to the northern part of Levant has been observed.

### Calabria: Warmer and drier

From the maps of linear trends in annual mean **temperature** for Europe compiled by Schönwiese and Janoschitz (2008) for Calabria a warming trend of about 1°C for the entire last century can by extracted, the value is consistent with the analysis by Gerstengarbe and Werner (2007), who compared the first and last decades of the 20th century. The respective value for the period from 1951 to 2000 is slightly larger than 0.6°C. This annual mean temperature trend does not reflect seasonality; warming was driven mainly by the summer months while for the winter months even a slight cooling trend was observed.

Overall it can be said that the most southern part of Italy and especially Calabria has become drier over the last decades. While the linear trend in annual **precipitation** for the entire 20th century for the Calabria region is almost zero, a pronounced trend exists for the period from 1951 to 2000 with a decrease in precipitation by about 20% in the annual mean with a decrease in summer precipitation of about 40% (Schönwiese and Janoschitz 2008). A comparison of the first three decades with the last three decades of the 20th century reveals a slightly drier Calabria at the end of the century (Gerstengarbe and Werner 2007). Luterbacher et al. (2006) analyzed winter precipitation anomalies for the last centuries in the Mediterranean region. The wettest decade was 1961 to 1970 and the driest was 1986 to 1995. The wettest (driest) multidecadal periods (30 winters in a row) were from 1951 to 1980 (1973 to 2002) with 5 mm (-15 mm) departures from the climatological average (1960 to 1990). Interestingly, in the overall wettest winters Calabria was drier than the climatological mean (10 to 20 mm) and in the overall driest winter series Calabria was about 10 to 20 mm wetter than the 61 to 90 average.

### Geographic and altitudinal distribution area of *Agonum viridicupreum*

**Figure 1.**
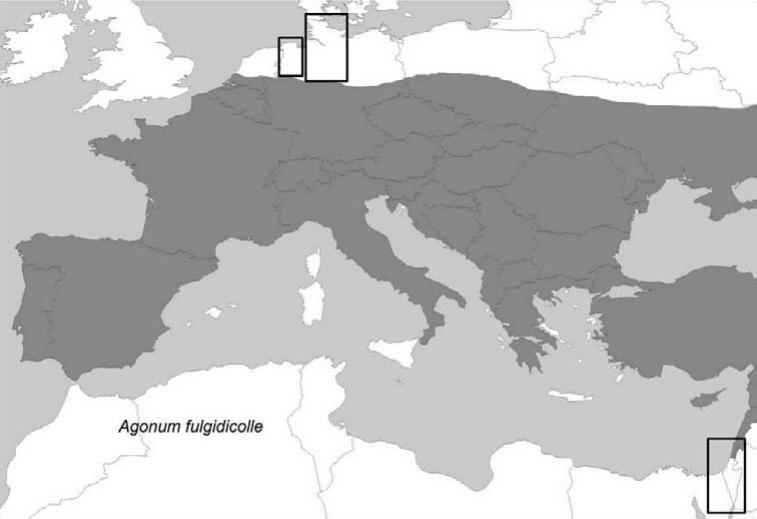
Distribution of *Agonum viridicupreum* (shaded in grey) and its sister taxon *Agonum fulgidicolle* in the western Palaearctic. Map modified after Turin et al. (2003) using information from Brandmayr et al. (2005) and personal observations. Frames indicate regions selected for more detailed analyses of records, see Figs 2 and 3.

**Figure 2.**
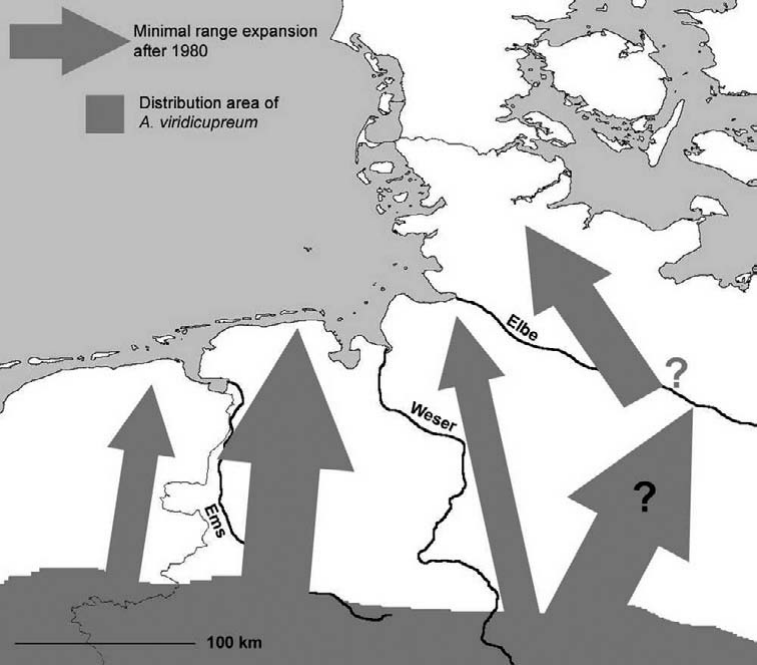
Distribution of *Agonum viridicupreum* (shaded in grey) in North-West Germany with eastern parts of the Netherlands. Arrows indicate minimum range expansion in the last three decades (for explanation and records see text). Range expansion in the Netherlands indicated after Turin, pers. comm.

The distribution area of the species within the western part of the Palaearctic is given in Fig. 1. *Agonum viridicupreum* occurs around the Mediterranean Sea (with a distribution gap in north-eastern Africa). The northern edge of the distribution area runs from the Netherlands through northern Germany and Poland (see also Fig. 2). In the south-east the species occurs in Turkey, Lebanon, and Israel.

In the southern Iberian Peninsula and Morocco, the beetle prefers mountainous areas (Zaballos and Jeanne 1994), but in the central and northern parts of Spain it also thrives well in lowland habitats (down to sea level, e.g. close to Oviedo, in the mountains up to about 2000 m a.s.l., CAB). In south-eastern Europe, the species occurs in mountains as well as lowlands (e.g. Peloponnese, CAB).

**Figure 3.**
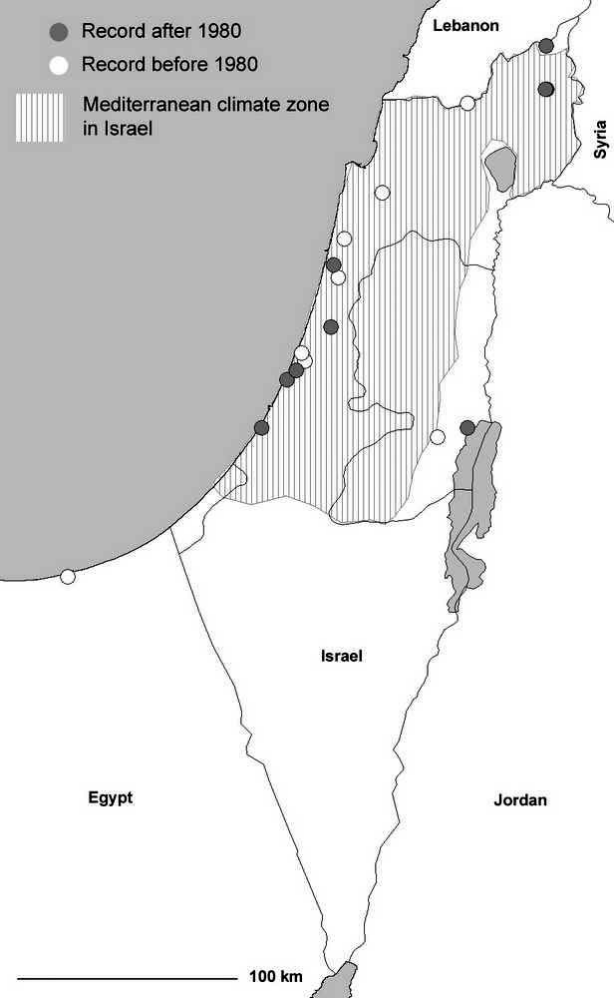
Distribution of *Agonum viridicupreum* in Israel. The striped area indicates Mediterranean climate zone (according to Yom-Tov and Tchernov 1988). Species’ records are taken from collections TAU, CAB and CWB.

The south-eastern distribution edge in the Levant virtually coincides with the border of the Mediterranean climate (Fig. 3). In this study, we report the first record for Egypt ([(T)El Arish, Sinai, leg. L. Fishelsohn, 12.03.1956], record in TAU, Fig. 3). However, the single specimen collected is not a proof of the existence of an autochthonous population here. The same is true for records obtained from the desert regions (e.g. Dead Sea Region, where no suitable habitats for the species occur, cf. Fig. 3).

### Faunistic analyses of the distribution margins

#### West Lower Saxony (west of the river Weser): 

Although *Agonum viridicupreum* has been known from the Netherlands since the 19th century, no specimens were recorded from West Lower Saxony until the 1980s (Table 1). Indeed, in the 1950s the northern distribution limit of the species’ range was located southwards of Lower Saxony, in the Westphalian Lowlands (Horion 1941; Barner 1954; Westhoff 1881). However, after 1981 numerous records from the whole Lower Saxonian (and Westphalian) Lowlands, northwards to the North Sea, were reported (Assmann and Ehrnsberger 1990; Mossakowski 1991; Handke and Kundel 1996; Fuellhaas 1998; Günther and Assmann 2005; Hannig and Schwerk 2000; Hannig 2001, 2005, 2008, numerous records in collections, e.g. CAB), thus, expanding the former northern distribution margin (Fig. 2). The distance between the known northern limit of the 19th century (central Westphalian Lowlands) and the present records close to the North Sea coast is more than 100 km (Fig. 2).

**Table 1.** Number of records of *Agonum viridicupreum* in the different periods of time in north-western Germany (leading edge) and Israel (rear edge).

*Study region*	*Number of records*		
	*before 1950*	*1950–1980*	*after 1980*
Leading edge:			
North-West Germany – West Lower Saxony	0	0	26
North-West Germany – East Lower Saxony and Schleswig Holstein	11	12	24
Rear edge:			
Israel	12		14

**1** Close to Geesthacht (leg. Kolze, 1890 [river Elbe, east of Hamburg], because of lacking records from the surrounding seen as diversion by Lohse, 1954) **2** Pevestorf [river Elbe, south-east of Lüneburg]

#### East Lower Saxony (east of river Weser):

Horion (1941) and Gersdorf and Kuntze (1957) listed records of the species from the hilly countryside close to Hannover. The latter authors questioned the occurrence of the species in the lowlands of eastern Lower Saxony. Along the river Elbe, one old record (19th century) is known from one site south-east of Hamburg (“?” in Fig. 2, Table 1). Lohse (1954) interpreted the presence of these specimens as vagrants transported downstream by flooding events from south-eastern Germany. However, these specimens could have originated also from temporal populations.

Between 1951 and 1980, only one record from another site in the Lower Saxonian floodplain area of the river Elbe is known (Table 1). Records from sites outside the given floodplain are exclusively known since 1981, when the number of records greatly increased.

Today, the species is found northwards, up to central Schleswig-Holstein (www.entomologie.de/hamburg/karten/fhl_02/_agovir1.htm), and reaches also the north-western parts of the considered area. Interpreting the old records from the floodplain area of the river Elbe as autochthonous populations leads us to think that the species’ geographic range has experienced a northward shift of about 100 km during the last century. Even if these records were not seen as autochthonous populations, the shift would have spanned over about 200 km (Fig. 2).

#### Levant:

Bodenheimer (1937) listed *Agonum viridicupreum* from Israel for the first time, and the former documented records were taken in the 1920s (TAU). In this region, the beetle is abundant at many rain or winter ponds (up to ca. 20 individuals per hour can be collected by hand picking; personal observations). So, it is very likely that the late discovery of the species at its south-eastern distribution edge would be a consequence of the poor carabidological exploration of the country. Since Bodenheimer’s time, numerous new records of the species have been reported, also during the last years (Fig. 3). *Agonum viridicupreum* reaches the south-eastern limit of the Mediterranean climate in Israel. There is no evidence for a northward shift of its distribution range, as the known southern Israeli populations are close to the semi-arid climate region from where the species is virtually unknown; only singletons – not indicating autochthonous populations – have been found (see above and Fig. 3).

#### Calabria:

For southern Italy (Calabria) there are scarce historical records (before 1980; Magistretti 1965; Angelini 1991). After 1980, a total of 37 specimens were recorded (Mazzei et al. 2010).

## Discussion

### Poleward shift of the leading edge

The compilation of the faunistic data showed that the distribution range of *Agonum viridicupreum* had significantly shifted northwards within the last 50 to 100 years. Up to 1950, in the analyzed region, the northern edge of the species’ distribution had stretched from the Netherlands (Nijmegen, Enschede; Turin 2000) southwards to the Westphalian Lowlands (south-western Lower Saxony), and again northwards to Hannover and Braunschweig (Fig. 1). The historical lack of the species studied in the region around Osnabrück (south-western Lower Saxony) does not need to be the result of undersampling, as also other thermophilous insects colonized this region later than the neighbouring western or eastern regions (e.g. several grasshopper species; Hochkirch 2001; Hochkirch and Damerau 2009).

Today, in the western part of Lower Saxony, *Agonum viridicupreum* can be found up to the North Sea, confirming a northwards range expansion of about 100 km. Similarly, in the neighbouring Netherlands the beetle has expanded its distribution range northwards and can nowadays be recorded close to the city of Groningen (Turin, pers. comm.). Also in the eastern part of Lower Saxony the species has spread northwards a minimum distance of 100 km and it reaches the centre of Schleswig-Holstein today. These results allow the assumption that a further temperature rise will make the species’ occurrence in Denmark highly probable in the near future.

### Stable rear edge

Unlike the northern distribution edge, the southern range margin (rear edge) of *Agonum viridicupreum* has not changed within the last decades. Indeed, there are still populations with numerous individuals south of Tel Aviv, which is close to the southern limit of the Mediterranean climate. Consequently, in Israel we expect the beetle to occur in most of the regions characterized by Mediterranean climate. In southern Italy (Calabria) *Agonum viridicupreum* shows a stable rear edge north of the 39th parallel, with permanent populations in the Crati Valley in the Cosenza province, in areas marked by Submediterranean or Mediterranean climate.

### Different behaviour of leading and rear edges

In accordance with numerous other authors (e.g. Hengeveld 1985; Hickling et al. 2006), we interpret the poleward shift of the leading edge of this species as a consequence of increasing temperature. It seems to be more difficult to describe the differences between leading and rear edge. In general, they could be explained by either intrinsic or extrinsic factors, or even a combination of both. The influence of *intrinsic* factors would imply that distinct genetic make-ups of the populations from the opposite edges of the distribution range are likely (Hampe and Petit 2005). However, so far there are no available investigations to corroborate this hypothesis for *Agonum viridicupreum* or other ground beetle species with the tendency of invasions in the Levant. Consequently, additional investigations are necessary. For non-migratory butterflies, it has been demonstrated that population size fluctuations are more pronounced at the leading edge than at the rear one (Parmesan et al. 1999) – a possible indication of less well-adapted populations at the leading edge. As our data do not give information about the population sizes of *Agonum viridicupreum* at the various sites, on the one hand, we cannot investigate this assumption. On the other hand, the high catching rates (which are comparable to catches from sites in northern Italy and Germany) do not support the assumption of the species declining in the Levant.

On the contrary, *extrinsic* factors may be acting differently at the leading and rear edge of the distribution range of *Agonum viridicupreum*. Our compilation of recent climate trends, however, reveals generally rising temperatures in all regions under study. In contrast, less consistent changes in precipitation can be observed. The populations in both, Calabria and the Levant will have to deal with a reduction in mean annual precipitation. In the face of the virtual exclusive occurrence of the species close to rain ponds in the southern edge of its distribution, it seems likely that the southern populations are limited by the given ground water tables which predominantly result from the annual amount of precipitation (mainly in the winter months).

In the Levant, larval development takes place during winter and early spring, as revealed by numerous tenerals, even at higher altitudes, e.g. 900 m a.s.l., in the Golan Heights, in April and May (personal observation). In contrast, the northern populations in Central Europe are unlikely to be limited by the amount of precipitation, but rather by temperatures during the species’ activity period. In fact, in this region, larval development takes place during summer and tenerals occur in late summer and autumn (August to October; Turin 2000; personal observations; during this season tenerals has never been found in the Levant). Finally, the role of other factors such as interactions with other organisms cannot be excluded when interpreting the distribution changes at the species’ range margins.

Nonetheless it is possible that the northern and southern limits of *Agonum viridicupreum* are determined by different climatic factors: increasing summer temperature in the north and increasing precipitation during the winter in the south can explain the poleward shift of the leading and the stable rear edge of the given species’ distribution.

### Potential of *Agonum viridicupreum* for further ecological research on global change

This study is the first one that investigates simultaneously the possible shifts of the northern and southern margins of a carabid species’ distribution due to climate change. Undoubtedly, at present we are not able to fully understand the underlying mechanisms of the different behaviours of the leading and rear boundaries of the geographic range of *Agonum viridicupreum* in the actual context of global change. However, our analysis suggests that the reaction of the study species to climate change may be more intricate than expected at first. For this reason, we think that the more complex situation in *Agonum viridicupreum* has important potential for further carabidological investigations at the interface of global change ecology and conservation biology. For instance, predictions based on climate envelope modelling, which has become both commonplace for many other animal species and the object of an intensive (and critical) scientific discourse (Settele et al. 2009; Rödder and Dambach 2010), can be optimized (and evaluated) by using the chronosequences of distribution data. To our knowledge, this approach has not yet been applied for ground beetles, although they appear to be an excellent object to validate climate envelope models, thanks to the outstanding faunistic work with numerous records from many regions and time periods (e.g. Luff 1998; Turin 2000; Desender et al. 2008; Trautner, in prep.).

## References

[B1] Alfieri A (1976) The Coleoptera of Egypt. Mémoires de la Société Entomologique de Égypte 5:1-361.

[B2] Angelini F (1991) Coleotterofauna dell’altipiano della Sila (Calabria, Italia) (Coleoptera). Memorie Società Entomologica Italiana 70:171-254.

[B3] Antoine M (1955f) Coléoptères carabiques du Maroc. Memoires de la Société des Sciences Naturelles et Physiques du Maroc, Zoologie 1ff: 1–692.

[B4] Assmann T (1991) Die ripikole Carabidenfauna der Ems zwischen Lingen und dem Dollart. Osnabrücker naturwissenschaftliche Mitteilungen 17:95-112.

[B5] Assmann T Dormann W Främbs H Gürlich S Handke K Huk T Sprick P Terlutter H (2003) Rote Liste der in Niedersachsen und Bremen gefährdeten Sandlaufkäfer und Laufkäfer (Coleoptera: Cicindelidae et Carabidae) mit Gesamtartenverzeichnis. Informationsdienst Naturschutz Niedersachsen 23:70-95.

[B6] Assmann T Ehrnsberger R (1990) Die Laufkäferfauna im Flurbereinigungsgebiet “Plaggenschale”. Osnabrücker naturwissenschaftliche Mitteilungen 16:39-50.

[B7] Austin K Small E Lemaire J-M Jeanne C Makris C Georghiou G (2008) Revision du Catalogue des Carabidae (Coleoptera) de Chypre. A revised catalogue of the Carabidae (Coleoptera) of Cyprus. Annales du Museum d‘Histoire Naturelle de Nice 23:1-199.

[B8] Bangsholt F (1983) Sandspringernes og lobebillernes udbredelse og forekomst i Danmark ca. 1830–1981. Scandinavian Science Press, Kobenhavn, 271 pp.

[B9] Barner K (1954) Die Cicindeliden und Carabiden der Umgebung von Minden und Bielefeld III. Abhandlungen aus dem Landesmuseum für Naturkunde zu Münster in Westfalen 16:1-64.

[B10] Ben-Gai T Bitan A Manes A Alpand P Rubin S (1999) Temporal and spatial trends of temperature patterns in Israel. Theoretical and Applied Climatology 64:163-177. doi: 10.1007/s007040050120

[B11] Blake S (2001)*Stenolophus mixtus* (Herbst) (Carabidae) new to Scotland. Coleopterist 10: 47.

[B12] Bobbink R Hornung M Roelofs JGM (1998) The effects of air-borne nitrogen pollutants on species diversity in natural and semi-natural European vegetation. Journal of Ecology 86:717-738. doi: 10.1046/j.1365-2745.1998.8650717.x

[B13] Bodenheimer FS (1937) Prodromus Faunae Palestinae. Essai sur les éléments zoogéographiques et historiques du Sud-Ouest du sous-règne paléarctique. Mémoires de l’Institute d’Égypte 33:1-286.

[B14] Bonadona P (1971) Catalogue des Coléoptères Carabiques de France. Nouvelle Revue d’Entomologie Suppl.:177-$5.

[B15] Bonn A (2000) Flight activity of carabid beetles on a river margin in relation to fluctuating water levels. In: Brandmayr P, Lövei G, Zetto Brandmayr T, Casale A, Vigna Taglianti A (Eds) Natural History and Applied Ecology of Carabid Beetles. Pensoft Publishers, Sofia, Moscow, 145–158.

[B16] Bonn A Hagen K Helling B (1997) Einfluß des Überschwemmungsregimes auf die Laufkäfer- und Spinnengemeinschaften in Uferbereichen der Mittleren Elbe und Weser. Arbeitsberichte Landschaftsökologie Münster 18:177-191.

[B17] Bonn A Hagen K Wohlgemuth-Von Reiche D (2002) The significance of flood regimes for carabid beetle and spider communities in riparian habitats - A comparison of three major rivers in Germany. River Research and Applications 18:43-64. doi: 10.1002/rra.632

[B18] Bousquet Y (2003) Tribe Platynini Bonelli, 1810. In: Löbl I, Smetana A (Eds) Catalogue of Palaearctic Coleoptera, Volume 1: Archostemata, Myxophaga, Adephaga. Apollo Books, Stenstrup, 449–469.

[B19] Brandmayr P Zetto T Pizzolotto R Casale A Vigna-Taglianti A (2005) I Coleotteri Carabidi per la valutazione ambientale e la conservazione della biodiversità. Agenzia per la protezione dell‘ambiente per i servizi tecnici. Manuali e Linee Guida, Rome, 240 pp.

[B20] Burakowski B Mroczkowski M Stefanska J (1973f) Coleoptera - Carabidae. Panstwowe Wydawnictwo Naukowe, Warszawa, 430 pp.

[B21] Casale A Vigna-Taglianti A (1999) Caraboid beetles (excl. Cicindelidae) of Anatolia, and their biogeographical significance (Coleoptera, Caraboidea). Biogeographia 20:277-406.

[B22] Curcic SB Brajkovic MM Curcic BPM (2007) The carabids of Serbia. Institute of Zoology, Faculty of Biology, University of Belgrade, Belgrad, 1083 pp.

[B23] Desender K Dekoninck W Maes D Crevecoeur L Dufrêne M Jacobs M Lambrechts J Pollet M Stassen E Thys N (2008) Een nieuwe verspreidingsatlas van de loopkevers en zandloopkevers (Carabidae) in België. Instituut voor Natuur- en Bosonderzoek, Brussel, 1–184 pp.

[B24] Drovenik B Peks H (1999) Catalogus faunae. Carabiden der Balkanländer. Coleoptera Carabidae. Coleoptera - Schwanfelder Coleopterologische Mitteilungen Sonderheft 1:1-123.

[B25] Fischer M Fuellhaas U Huk T (1998) Laufkäferzönosen unterschiedlich anthropogen beeinflußter Feuchtgrünländer in vier Niedermooren Norddeutschlands. Angewandte Carabidologie 1:13-22.

[B26] Fuellhaas U (1997) Der Einfluß von Vernässung und Überstauungsmaßnahmen in degeneriertem Niedermoorgrünland auf ausgewählte Laufkäferarten (Coleoptera: Carabidae). Arbeitsberichte Landschaftsökologie Münster 18:133-146.

[B27] Fuellhaas U (1998) Restitution von Feuchtgrünland auf Niedermoor - Der Einfluss mehrjähriger Überstau- und Vernässungsmaßnahmen auf Laufkäferzönosen. Angewandte Carabidologie 1:4-12.

[B28] Gersdorf E Kuntze K (1957) Zur Faunistik der Carabiden Niedersachsens. Berichte Naturhistorische Gesellschaft Hannover 103:101-136.

[B29] Gerstengarbe F-W Werner PC (2007) Der rezente Klimawandel. In: Endlicher W, Gerstengarbe F-W (Eds) Der Klimawandel - Einblicke, Rückblicke und Ausblicke. Potsdam-Institut für Klimafolgenforschung und Humboldt-Universität zu Berlin, Berlin, 34–43.

[B30] Gobbi M De Bernardi F Pelfini M Rossaro B Brandmayr P (2006) Epigean arthropod succession along a 154-year glacier foreland chronosequence in the Forni Valley (Central Italian Alps). Arctic, Antarctic, and Alpine Research 38:357-362. doi: 10.1657/1523-0430(2006)38[357:EASAAY]2.0.CO;2

[B31] Gobbi M Rossaro B Vater A De Bernardi F Pelfini M Brandmayr P (2007) Environmental features influencing Carabid beetle (Coleoptera) assemblages along a recently deglaciated area in the Alpine region. Ecological Entomology 32:682-689. doi: 10.1111/j.1365-2311.2007.00912.x

[B32] Guérguiev VB Guérguiev BV (1995) Catalogue of the ground-beetles of Bulgaria (Coleoptera: Carabidae). Pensoft, Sofia, 279 pp.

[B33] Günther J Assmann T (2005) Restoration ecology meets carabidology: effects of floodplain restitution on ground beetles (Coleoptera, Carabidae). Biodiversity and Conservation 14:1583-1606. doi: 10.1007/s10531-004-0531-4

[B34] Gürlich S Suikat R Ziegler W (1995) Katalog der Käfer Schleswig-Holsteins und des Niederelbegebietes. Verhandlungen des Vereins für Naturwissenschaftliche Heimatforschung zu Hamburg eV 41:1-111.

[B35] Hampe A Petit RJ (2005) Conserving biodiversity under climate change: the rear edge matters. Ecology Letters 8:461-467. doi: 10.1111/j.1461-0248.2005.00739.x 21352449

[B36] Handke K (1995) Zur Laufkäferfauna eines Bremer Flußmarschengebietes (Niedervieland/Ochtumniederung/Ochtumsand). Zeitschrift für Ökologie und Naturschutz 4:203-225.

[B37] Handke K Kundel W (1996) Veränderungen der Vegetation und Fauna auf überstauten Grünlandflächen im Niedervieland - Ergebnisse sechsjähriger Untersuchungen im GVZ-Ausgleichsraum. Bremer Beiträge für Naturkunde und Naturschutz 1:179-187.

[B38] Hannig K (2001) Faunistische Mitteilungen über ausgewählte Laufkäferarten (Col. Carabidae) in Westfalen, Teil IV. Natur und Heimat 61:97-110.

[B39] Hannig K (2004) Aktualisierte Checkliste der Sandlaufkäfer und Laufkäfer (Coleoptera: Cicindelidae, Carabidae) Westfalens (Bearbeitungsstand: 31.01.2003). Angewandte Carabidologie 6:71-86.

[B40] Hannig K (2005) Faunistische Mitteilungen über ausgewählte Laufkäferarten (Col., Carabidae) in Westfalen, Teil VI. Natur und Heimat 65:49-60.

[B41] Hannig K (2008) Faunistische Mitteilungen über ausgewählte Laufkäferarten (Col., Carabidae) in Nordrhein-Westfalen II. Natur und Heimat 68:53-64.

[B42] Hannig K Schwerk A (2000) Faunistische Mitteilungen über ausgewählte Laufkäferarten (Col., Carabidae) in Westfalen, Teil II. Natur und Heimat 60:15-24.

[B43] Hengeveld R (1985) Dynamics of Dutch ground beetle species during the twentieth century. Journal of Biogeography 12:389-411. doi: 10.2307/2844950

[B44] Hengeveld R (1989) Dynamics of species invasions. Chapman & Hall, London and New York, 160 pp.

[B45] Hickling R Roy DB Hill JK Fox R Thomas CD (2006) The distributions of a wide range of taxonomic groups are expanding polewards. Global Change Biology 12:450-455. doi: 10.1111/j.1365-2486.2006.01116.x

[B46] Hieke F Wrase DW (1988) Faunistik der Laufkäfer Bulgariens (Coleoptera, Carabidae). Deutsche Entomologische Zeitschrift 35:1-171. doi: 10.1002/mmnd.19880350102

[B47] Hochkirch A (2001) Rezente Areal- und Bestandsveränderungen bei Heuschrecken Nordwestdeutschlands (Orthoptera, Saltatoria). Verhandlungen des Westdeutschen Entomologen Tages 2000:167-178.

[B48] Hochkirch A Damerau M (2009) Rapid range expansion of a wing-dimorphic bush-cricket after the 2003 climatic anomaly. Biological Journal of the Linnean Society 2009:118-127. doi: 10.1111/j.1095-8312.2008.01199.x

[B49] Horion A (1941) Faunistik der deutschen Käfer I. Hans Goecke Verlag, Krefeld, 463 pp.

[B50] Hurka K (1996) Carabidae of the Czech and Slovak Republics. Kabourek, Zlín, 565 pp.

[B51] IPCC (2007) Climate change 2007: impacts, adaptation and vulnerability. In: Parry ML, Canziani OF, Palutikof JP, van der Linden PJ, Hanson CE (Eds) Contribution of working group II to the fourth assessment report of the intergovernmental panel on climate change Cambridge University Press, Cambridge, 976 pp.

[B52] Irmler U Gürlich S (2004) Die ökologische Einordnung der Laufkäfer (Coleoptera: Carabidae) in Schleswig-Holstein. Faunistisch-Ökologische Mitteilungen Supplement 32:1-117.

[B53] Jacobeit J Dünkeloh A Hertig E (2007) Mediterranean rainfall changes and their causes. In: Lozán J, Graßl H, Hupfer P, Menzel L, Schönwiese C-D (Eds) Global Change: Enough water for all? , Hamburg, 195–199.

[B54] Jeanne C Zaballos JP (1986) Catalogue des Coleopteres Carabiques de la Penninsule Iberique. Bulletin de la Société Linnéenne de Bordeaux Supplement: 1–186.

[B55] Jeannel R (1941f) Coléoptères Carabiques. Lechevalier, Paris, 1173 pp.

[B56] Khatib I Gerstengarbe F-W Haj-Daoud A (2007) East Mediterranean climate change trends in the last century. Arab Water World (AWW) Vol. XXXI: 9 6 pp.

[B57] Kocher L (1963) Catalogue commenté des Coléoptères du Maroc. Travaux de l‘Institut Scientifique Chérifien 27:1-170.

[B58] Köhler F Klausnitzer BEds (1998) Verzeichnis der Käfer Deutschlands. Dresden, 185 pp.

[B59] Kryzhanovskij OL Belousov IA Kabak II Kataev BM Makarov KV Shilenkov VG (1995) A checklist of the ground-beetles of Russia and adjacent lands (Insecta, Coleoptera, Carabidae). Pensoft, Sofia, Moscow, 271 pp.

[B60] Kvamme T (1978) *Stenolophus mixtus* Hbst., an expanding carabid beetle new to Norway. Norwegian Journal of Entomology 25:227-228.

[B61] Lindroth CH (1972) Changes in the Fennoscandian ground-beetle fauna (Coleoptera, Carabidae) during the twentieth century. Annales Zoologici Fennici 9:49-64.

[B62] Lindroth CH (1985) The Carabidae (Coleoptera) of Fennoscandia and Denmark. Fauna Entomologica Scandinavica 15:1-225.

[B63] Lohse G-A (1954) Die Laufkäfer des Niederelbegebietes und Schleswig-Holsteins. Verhandlungen des Vereins für Naturwissenschaftliche Heimatforschung zu Hamburg 31:1-39.

[B64] Luff ML (1998) Provisional atlas of the ground beetles (Coleoptera, Carabidae) of Britain. Biological Records Centre, Huntingdon, 194 pp.

[B65] Luff ML (2007) The Carabidae (ground beetles) of Britain and Ireland. Handbooks for the Identification of British Insects 4, Part 2:1-247.

[B66] Luterbacher J Xoplaki E Casty C Wanner H Pauling A Kuettel M Rutishauser T Broennimann S Fischer E Fleitmann D Gonzalez-Rouco JF García-Herrera R Barriendos M Rodrigo FS Gonzalez-Hidalgo JC Saz MA Gimeno L Ribera P Brunet M Paeth H Rimbu N Felis T Jacobeit J Duenkeloh A Zorita E Guiot J Turkes M Alcoforado MJ Trigo R Wheeler D Tett S Mann ME Touchan R Shindell DT Silenzi S Montagna P Camuffo D Mariotti A Nanni T Brunetti M Maugeri M Zerefos C Zolt SD Lionello P Nunes MF Rath V Beltrami H Garnier E Ladurie ELR (2006) Mediterranean climate variability over the last centuries: A review. In: Lionello P, Malanotte-Rizzoli P, Boscolo R (Eds) The Mediterranean Climate: an overview of the main characteristics and issues. Elsevier, Amsterdam, 27–148.

[B67] Machard P (1997) Catalogue des Coleopteres Carabiques du Maroc. Machard, Molineuf, 54 pp.

[B68] Magistretti M (1965) Coleoptera: Cicindelidae, Carabidae - Catalogo topografico. Edizioni Calderini, Bologna, 512 pp.

[B69] Marggi W (1992) Faunistik der Sandlaufkäfer und Laufkäfer der Schweiz (Cicindelidae & Carabidae). Text und Verbreitungskarten. Documenta Faunistica Helvetiae 13/1 and 13/2: 1–477 and 471–243.

[B70] Marggi W Luka H (2001) Laufkäfer der Schweiz. Gesamtliste 2001. Opuscula Biogeographica Basileensia 1:1-37.

[B71] Mazzei A Bonacci T Sapia M Brandmayr P (2010) La carabidofauna dell’ecotopo fluviale del Crati (Cosenza, Italia) (Coleoptera, Carabidae). Naturalista Siciliano, IV: 185–198

[B72] Mossakowski D (1991) Zur Verbreitung der Laufkäfer im Lande Bremen. Abhandlungen des Naturwissenschaftlichen Verein zu Bremen 41:543-640.

[B73] Müller-Motzfeld G (Ed) (2004) Bd. 2 Adephaga 1: Carabidae (Laufkäfer). Spektrum, München, 521 pp.

[B74] Neculiseanu ZZ Matalin AV (2000) A catalogue of the ground beetles of the Republic of Moldova (Insecta, Coleoptera: Carabidae). Pensoft, Sofia, 164 pp.

[B75] Nitzu E (1997) Carabidae (Coleoptera) from Israel. Travaux de l‘institut de Spéologie 'Émile Racovitza' 36:99-106.

[B76] Palm T (1982) Förändringar i den svenska skalbaggsfaunan. Ent Tidskr 103:25-32.

[B77] Parmesan C (2006) Ecological and evolutionary responses to recent climate change. Annual Review of Ecology and Systematics 37:637-669. doi: 10.1146/annurev.ecolsys.37.091305.110100

[B78] Parmesan C Ryrholm N Stefanescu C Hill JK Thomas CD Descimon H Huntley B Kaila L Kullberg J Tammaru T Tennent WJ Thomas JA Warren M (1999) Poleward shifts in geographical ranges of butterfly species associated with regional warming. Nature 399:579-583. doi: 10.1038/21181

[B79] Pauli H Gottfried M Reier K Klettner C Grabherr G (2007) Signals of range expansions and contractions of vascular plants in the high Alps: observations (1994–2004) at the GLORIA*master site Schrankogel, Tyrol, Austria. Global Change Biology 13:147-156. doi: 10.1111/j.1365-2486.2006.01282.x

[B80] Puel L (1938) Les *Agonum* paléarctiques. Miscellanea Entomologica 39:157-200.

[B81] Quante M (2010) The changing climate: Past, Present, Future. In: Habel JC, Assmann T (Eds) Relict Species: Phylogeography and Conservation Biology. Springer, Heidelberg, 9–56.

[B82] Rödder D Dambach J (2010) Review: Modelling Future Trends of Relict Species. In: Habel JC, Assmann T (Eds) Relict Species: Phylogeography and Conservation Biology. Springer, Heidelberg, 373–384.

[B83] Saaroni H Ziv B Alpert P (2003) Long-term variations in summer temperatures over the eastern Mediterranean. Geophysical Research Letters 30: 1946. doi: 10.1029/2003GL017742

[B84] Schlünzen KH Hoffmann P Rosenhagen G Riecke W (2010) Long-term changes and regional differences in temperature and precipitation in the metropolitan area of Hamburg. International Journal of Climatology 30:1121-1136.

[B85] Schönwiese C-D Janoschitz R (2008) Klima-Trendatlas Deutschland 1901–2000. Berichte des Instituts für Atmosphäre und Umwelt der Universität Frankfurt/Main 7: 82.

[B86] Schuldt A Assmann T (2009) Environmental and historical effects on richness and endemism patterns of carabid beetles in the western Palaearctic. Ecography 32:705-714. doi: 10.1111/j.1600-0587.2009.05763.x

[B87] Serrano J (2003) Catalogue of the Carabidae (Coleoptera) of the Iberian Peninsula. Monografias SEA 9:1-130.

[B88] Settele J Kudrna O Harpke A Kühn I Swaay Cv Verovnik R Warren M Wiemers M Hanspach J Hickler T Kühn E Halder Iv Veling K Vliegenthart A Wynhoff I Schweiger O (2009) Climatic Risk Atlas of European Butterflies. Biorisk 1:1-710.

[B89] Silfverberg H (2005) Newcomers in the coleopteran fauna of northern Europe. In: Sklodowski J, Huruk S, Bersevskis A, Tarasiuk S (Eds) Protection of Coleoptera in the Baltic Sea region. Warsaw Agricultural University Press, Warsaw, 93–101.

[B90] Steinberger EH Gazit-Yaari N (1996) Recent changes in the spatial distribution of annual precipitation in Israel. Journal of Climate 9:3328-3336. doi: 10.1175/1520-0442(1996)009<3328:RCITSD>2.0.CO;2

[B91] Turin H (2000) De Nederlandse Loopkevers - Verspreiding en oecologie. Nationaal Natuurhistorisch Museum Naturalis, Leiden, 666 pp.

[B92] Turin H Penev L Casale AEds (2003) The Genus *Carabus* in Europe - a Synthesis. Pensoft Publishers & European Invertebrate Survey, Sofia, Moscow & Leiden, 511 pp.

[B93] Westhoff F (1881) Die Käfer Westfalens I. Verhandlungen des naturhistorischen Vereins der preussischen Rheinlande und Westfalens Supplement 38.

[B94] Westhoff F (1882) Die Käfer Westfalens II. Verhandlungen des naturhistorischen Vereins der preussischen Rheinlande und Westfalens Supplement 38.

[B95] Wrase DW (2009) New or interesting records of carabid beetles from Europe, Madeira, northern Africa, Turkey, from the Near East, Iran, Iraq, Kuwait, and Pakistan, with nomenclatorial and taxonomic notes (Coleoptera, Carabidae, Bembidiini, Brachinini, Cyclosomini, Elaphrini, Harpalini, Lebiini, Nebriini, Platynini, Pterostichini, Scaritini, Sphodrini, Zabrini). Linzer Biologische Beitraege 41:901-935.

[B96] Yom-Tov Y Tchernov E (1988) The Zoogeography of Israel: The Distribution and Abundance at a Zoogeographical Crossroad (Monographiae Biologicae). Dr. W. Junk Publishers, Dordrecht / Boston / Lancaster, 616 pp.

[B97] Zaballos JP Jeanne C (1994) Nuevo catalogo de los carabidos (Coeloptera) de la Peninsula Iberica. Monografias SEA 1:1-159.

[B98] Ziegler W (1997) Vierter Nachtrag zur Käferfauna von Schleswig-Holstein und dem Niederelbegebiet. Bombus 3:92-102.

[B99] Ziegler W (2004) Sechster Nachtrag zur Käferfauna Schleswig-Holsteins und des Niederelbegebietes. Bombus 3:243-252.

